# Is self-perception related to speech perceptual analysis measures in early- and late-onset Parkinson’s disease?

**DOI:** 10.1590/1980-5764-DN-2024-0192

**Published:** 2025-09-22

**Authors:** Vanessa Brzoskowski dos Santos, Amanda Lara Bressanelli, Fernanda Zardin, Rui Rothe-Neves, Maira Rozenfeld Olchik

**Affiliations:** 1 Universidade Federal do Rio Grande do Sul, Ciências Médicas, Programa de Pós-Graduação em Medicina, Porto Alegre RS, Brazil.; 2 Universidade Federal do Rio Grande do Sul, Curso de Fonoaudiologia, Porto Alegre RS, Brazil.; 3 Universidade Federal de Minas Gerais, Faculdade de Letras, Laboratório de Fonética, Belo Horizonte MG, Brazil.; 4 Universidade Federal do Rio Grande do Sul, Departamento de Cirurgia e Ortopedia, Porto Alegre RS, Brazil.

**Keywords:** Parkinson Disease, Speech, Dysarthria, Speech Disorders, Self Concept, Doença de Parkinson, Fala, Disartria, Distúrbios da Fala, Autoimagem

## Abstract

Dysarthria is prevalent in Parkinson’s disease (PD) and has negative effects on communication and social interaction. A gap exists in understanding whether patient-reported outcome measures can effectively identify speech disorders prematurely.

**Objective:**

Compare the relationship between patients’ reported outcome measures and the speech objective outcome measure between early-onset (EOPD) and late-onset Parkinson’s disease (LOPD).

**Methods:**

Thirty-nine participants diagnosed before age 50 (EOPD) and 32 diagnosed older than 50 (LOPD) were included. Self-perception was collected through the Radboud Oral Motor Inventory for Parkinson’s Disease questionnaire. The speech samples collected involved diadochokinesia (/pataka/) and monologue. Auditory perceptive analysis was performed through the degree of dysarthria and acoustic analysis of speech.

**Results:**

Regarding speech characterization, there were also no significant differences in ROMP (p=0.462), degree of dysarthria (p=0.423), and acoustic variables. In EOPD, the ROMP self-perception scale was consistent with the degree of dysarthria assigned by professionals through perceptual auditory analysis. However, in LOPD, there were discrepancies when patients had a severe degree of dysarthria. Additionally, both groups had a positive and significant correlation between disease duration and the degree of dysarthria concerning ROMP

**Conclusion:**

The results between clinical diagnosis of dysarthria and self-perception in the early and late onset groups showed no significant differences, indicating that self-perception may be a useful tool in identifying dysarthria.

## RESUMO.

A disartria é prevalente na doença de Parkinson (DP) e tem efeitos negativos na comunicação e interação social. Existe uma lacuna na compreensão sobre se as medidas de resultados relatados pelos pacientes podem identificar com eficácia os distúrbios da fala prematuramente.

**Objetivo:**

Comparar a relação entre as medidas de resultados relatadas pelos pacientes e a medida de resultados objetivos de fala entre a doença de Parkinson de início precoce (EOPD) e tardio (LOPD).

**Métodos:**

Foram incluídos 39 participantes com EOPD e 32 com LOPD. A autopercepção foi coletada por meio do questionário Radboud Oral Motor Inventory for Parkinson’s Disease. As amostras de fala coletadas envolveram diadococinesia (/pataka/) e monólogo. Foi realizada análise perceptiva auditiva por meio do grau de disartria e análise acústica da fala.

**Resultados:**

Com relação à caracterização da fala não houve diferenças significativas no ROMP (p=0,462), grau de disartria (p=0,423) e variáveis acústicas. Na EOPD, a escala de autopercepção do ROMP foi condizente com o grau de disartria atribuido pelos profissionais por meio da análise perceptivo-auditiva. No entanto, no LOPD, houve discrepâncias quando os pacientes apresentavam grau severo de disartria. Além disso, ambos os grupos tiveram correlação positiva e significativa entre a duração da doença e o grau de disartria em relação à ROMP.

**Conclusão:**

Os resultados entre o diagnóstico clínico de disartria e a autopercepção nos grupos de início precoce e tardio não mostraram diferenças significativas, demonstrando que a autopercepção pode ser uma ferramenta útil na identificação da disartria.

## INTRODUCTION

 While the prevalence of dysarthria varies across studies, a significant majority of individuals with Parkinson’s disease PD are anticipated to encounter some degree of dysarthria during disease progression, ranging from 51 to 90%^1-4^. Hypokinetic dysarthria is the specific speech impairment most commonly associated with PD. It can affect respiration, phonation, articulation, and prosody, negatively impacting communicative and social interactions. In particular, the changes to speech have been associated with disrupted self-image, and the impact of speech changes comes before changes in intelligibility and affects individual and family life^5^. Moreover, the frustration and effort required to overcome communicative limitations may lead to social withdrawal and isolation^6,7^. 

 It is uncertain whether the dysarthria of PD and, by extension, the self-perception of speech difficulties differs between early-onset PD (EOPD) and late-onset PD (LOPD). The International Parkinson and Movement Disorder Society (MDS) defines EOPD as an onset up to 50, while LOPD has an onset after 50^8^. There are known differences between the phenotype-specific characteristics of EOPD and LOPD. People with EOPD tend to have slower progression, a higher cumulative incidence of dyskinesia, a longer threshold of wearing off, and greater on-off effects than those with LOPD^9,10^. EOPD has been shown to result in worse gait features, while LOPD is associated with worse posture features^11^. Additionally, people with EOPD tend to experience higher levels of disability related to work activities. In contrast, people with LOPD tend to exhibit worse cognitive abilities^12^ and higher levels of disability related to cognition^13^. 

 Speech differences between EOPD and LOPD need to be more understood. In a study by Rusz et al.^14^, speakers with EOPD demonstrated weaker inspirations, reflecting quieter voice, while speakers with LOPD exhibited worse voice quality and articulatory precision. Speech differences between EOPD and LOPD are likely the result of aging on neurodegeneration. Moreover, LOPD may have reduced compensatory mechanism capacity, resulting in more pronounced impairment and a faster progression. Dysarthria is present in EOPD and LOPD with prosodic and articulatory impairments. LOPD may exhibit more pronounced speech alterations^14,15^. Given the collective differences between EOPD and LOPD, it is reasonable to assume that self-perception of dysarthria may differ between these groups^1^. Discovering how accurate this self-awareness is could be a key clinical measure for early rehabilitation intervention^1^. 

 The assessment of self-perception of speech changes in PD is often completed using patient-reported outcome measures, such as the Radboud Oral Motor Inventory for Parkinson’s disease (ROMP). For the ROMP, scores range from 7 (normal) to 35 (severe impact) and reflect changes across different domains^16,17^. The ROMP’s internal consistency and test-retest reliability indicate a valid and reliable questionnaire^16,18^. Other measures of self-perception of dysarthria impact are not specifically tailored to individuals with PD, such as Living with Dysarthria^19^, the Dysarthria Impact Profile^20^, and the self-rated Speech Scale (SRSS)^21^. 

 Considering that LOPD presents with more expressed gait disturbances^22^, we may hypothesize that specific speech abnormalities will be detectable only in LOPD. However, the connection between these measures and individuals’ self-perception, especially in the context of differences between EOPD and LOPD Parkinson’s disease patients, remains to be elucidated. Therefore, this study aimed to compare the relationship between patients’ reported outcome measures of self-perception of speech and objective measures in speech across EOPD and LOPD. 

## METHODS

Observational cross-sectional study.

### Participants

 Individuals diagnosed with Parkinson’s disease from the Movement Disorders Outpatient Clinic of the Neurology Service from a reference hospital in the south region of Brazil were included in the study. All participants had Brazilian Portuguese as their native language. Patients diagnosed before age 50 were considered EOPD (n=39), while the age of onset older than 50 was classified as LOPD (n=32) according to recent expert consensus^8^. All participants signed an Informed Consent Form. Participants with a history of other neurological events, sensory or motor disorders, systemic diseases, and/or structural changes that affected the voice and/or speech were excluded. 

### Data collection

#### Clinical and sociodemographic data

 Gender, age, education level, duration of illness, and age at onset of symptoms were collected at the time of evaluation. The motor symptoms were evaluated through Movement Disorder Society-sponsored revision of the Unified Parkinson’s Disease Rating Scale (MDS-UPDRS)^23^ and the Hoehn & Yahr (H&Y) scale^24^ by a neurologist. 

#### Self-perception assessment

 The ROMP^16,17^ (Supplementary Material) is a self-administered questionnaire to verify patients’ self-perception of speech, swallowing, and saliva control. We only considered the speech domain for this study. The questionnaire delves into various aspects of speech and comprises seven questions, ranging from voice perception to the discomfort experienced by the patient due to speech difficulties. Respondents rate the frequency of symptoms on a scale of 1 to 5 for each item (1=normal; 5=worst score), with a maximum score of 35 suggesting self-perceived speech-related problems. 

### Speech data

 In a quiet environment without acoustic isolation, speech recording was performed in a single session using a digital tape recorder and a headset microphone approximately 5 cm from the patient’s mouth. The recordings were sampled at 44.1 kHz and quantified at 16 bits. The patients were asked to perform two tasks after a model provided by the researcher:

- diadochokinesis /pataka/ as fast as possible in a single breath;
- sixty seconds of spontaneous speech answering the question: "What have you done today since you have been awake?".

### Auditory perceptual analysis

 Auditory-perceptual analysis (APA) is the gold standard for dysarthria assessment^25-28^. Three speech therapists with at least seven years of experience completed an evaluation while blinded to group membership (EOPD and LOPD). Following a brief auditory training session, the therapists were presented with the recordings of the two tasks in randomized order. The degree of dysarthria was based on the five subsystems of speech (phonation, articulation, resonance, prosody, and respiration), and rated severity using a four-point scale (0=normal, 1=mild dysarthria, 2=moderate dysarthria, 3=severe dysarthria) based on Duffy’s definitions^28^. Excellent interrater agreement was achieved, with a Kappa Index coefficient≥0.90. 

### Acoustic speech analysis

 PRAAT software (version 6.1.55) was employed for acoustic speech analysis. An automated script was utilized to detect intensity peaks, with the count of these peaks serving as an equivalent measure for determining the number of syllables in Brazilian Portuguese, where only vowels are allowed in the core syllable. Demonstrating robust reliability relative to manual acoustic analysis^29^, this script adheres to parameters outlined by Rusz et al.^30^. Dependent variables for the diadochokinesis (DDK) and monologue tasks were the number of syllables, average syllable duration (ASD), and pause ratio (pause time divided by total duration). These acoustic variables were analyzed to characterize the sample and as a complement to the auditory perceptual analysis 

### Statistical analysis

 The independent variables (sex, age, education, disease duration, age at onset of symptoms, ROMP, H&Y, and UPDRS) were presented as descriptive analyses (absolute and relative frequencies, mean and standard deviation). To compare the degree of dysarthria between groups, the chi-square test was used. Statistical tests were selected according to the distribution data provided by the Kolmogorov-Smirnov and Shapiro-Wilk tests. Spearman’s test performed correlations between patient self-perception (ROMP) and objective measures (clinical data and speech features). The self-perception and the degree of dysarthria of individuals with EOPD and LOPD were compared using analysis of orthogonal contrasts variance. Statistical significance was defined as p<0.05. The statistical software used was the Statistical Package for the Social Sciences (SPSS) version 22.0. 

## RESULTS

 Seventy-one individuals with Parkinson’s Disease were included in the study, of which 39 (54.92%) had EOPD and 32 (45.07%) had LOPD. There were no significant differences between the groups regarding sex, years of education, H&Y scale, and UPDRS. Table 1 shows the sociodemographic data of the individuals in each group. 

**Table 1:** Sociodemographic data.

	EOPD (n=39)	LOPD (n=32)	p-value
Gender (male)	25 (64.1%)	18 (56.3%)	0.501
Age (years)	56.48 (±7.91)	67.5 (±7.79)	<0.001
Years of education	8.72 (±4.46)	9.03 (±4.08)	0.563
Disease Duration	14.64 (±6.7)	9.78 (±4.8)	0.002
Age of onset	41.84 (±6.42)	57.87 (±6.69)	<0.001
HY	1 (1-3)	2 (1-4)	0.808
MDS -UPDRS	15.85 (±10.22)	17.88 (±12.72)	0.561

Abbreviations: HY, Hoehn and Yahr Staging Scale of Disability; UPDRS, Unified Parkinson’s Disease Rating Scale; EOPD, Early-onset Parkinson’s Disease; LOPD, Late-onset Parkinson’s Disease.

Table 2 presents the speech characterization of the sample in each group. No statistically significant differences were observed between the groups regarding speech self-perception using the ROMP scale (p=0.462). The perceptual auditory analysis found no differences between the groups regarding degree of dysarthria (p=0.423). The same was observed in the variables of the acoustic analysis, indicating that the groups do not differ in terms of speech characteristics and self-perception. 

**Table 2:** Speech characteristics of the sample in each group in the EOPD and LOPD groups.

		EOPD (n=39)	LOPD (n=32)	p-value
ROMP		15.79 (±6.82)	14.43 (±6.35)	0.462
Dysarthria Degree	Mild	28 (71.8%)	19 (59.4%)	0.423
	Moderate	7 (13.7%)	10 (31.3%)	
	Severe	4 (7.8%)	3 (9.4%)	
DDK—number of syllables		35.79 (±24.61)	35.18 (±20.27)	0.698
DDK—ASD		0.208 (±0.056)	0.210 (±0.060)	0.238
Monologue—ASD		0.265 (±0.113)	0.268 (±0.059)	0.216
Monologue—pause ratio		0.296 (±0.190)	0.350 (±0.187)	0.169

Abbreviations: ROMP, Radboud Oral Motor Inventory for Parkinson’s Disease; DDK, diadochokinesis; ASD, average syllable duration.

Table 3 shows the correlation between ROMP, clinical data and the degree of dysarthria. Both groups showed a positive correlation between ROMP and disease duration: EOPD (longer disease duration) Rho=0.341 (weak correlation) and for LOPD (shorter disease duration) Rho=0.529 (moderate correlation). The same positive and significant correlation is observed in the groups in relation to ROMP and the degree of dysarthria: EOPD (Rho=0.529) moderate correlation and LOPD (Rho<0.622) moderate correlation. Furthermore, in the EOPD group, a weak and negative correlation was also observed between ROMP vs. years of education (Rho=-0.471) and a moderate and positive correlation between ROMP vs. UPDRS (Rho=0.510). In the LOPD group, a moderate and positive correlation was observed between ROMP vs. HY (Rho=0.516). 

**Table 3:** Correlation between Radboud Oral Motor Inventory for Parkinson’s Disease, clinical data, and degree of dysarthria in the early- and late-onset Parkinson’s desease groups.

	EOPD (n=39)		LOPD (n=32)	
	ROMP		ROMP	
	p-value	r	p-value	r
Gender (male)	0.470	0.119	0.766	-0.055
Age (years)	0.061	0.303	0.056	0.341
Years of education	0.002	-0.471	0.117	-0.292
Disease Duration	0.033	0.341	0.002	0.529
Disease onset	0.884	-0.024	0.933	-0.016
HY	0.210	0.249	0.020	0.516
UPDRS	0.018	0.510	0.523	0.167
Degree of dysarthria	0.001	0.529	<0.000	0.622

Abbreviations: ROMP, Radboud Oral Motor Inventory for Parkinson’s Disease; HY, Hoehn and Yahr Staging Scale of Disability; UPDRS, Unified Parkinson’s Disease Rating Scale.

**Figure 1:**
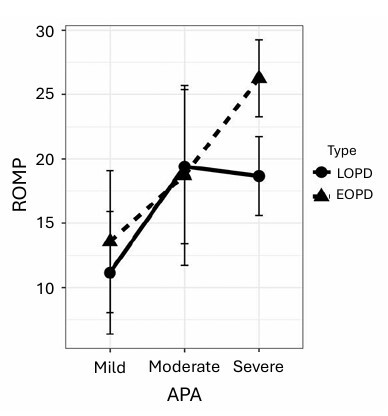
Comparison between self-perception and the degree dysarthria in the early- and late-onset Parkinson’s disease groups. Abbreviations: ROMP, Radboud Oral Motor Inventory for Parkinson’s Disease; EOPD, Early-onset Parkinson’s Disease; LOPD, Late-onset Parkinson’s disease; APA, Auditory Perceptual Analysis.

Figure 1 compares self-perception and degree of dysarthria in the EOPD and LOPD groups. In the EOPD group, it can be observed that the ROMP self-perception scale was consistent with the degree of dysarthria assigned by professionals through perceptual auditory analysis. However, in the LOPD group, there were discrepancies when the patient had a severe degree of dysarthria. This shows that individuals in the LOPD group with severe dysarthria assigned a lower score on the self-perception scale. 

## DISCUSSION

 In this study, we observed that, in the EOPD group, the ROMP self-perception scale aligned with the degree of dysarthria. However, in the LOPD group, discrepancies emerged when patients had severe dysarthria. Furthermore, we observed a positive correlation (weak for EOPD and moderate for LOPD) between ROMP scores and disease duration in both groups. Both groups also found a positive and moderate correlation between ROMP scores and degree of dysarthria. This indicates that the higher the score on the self-perception scale, the higher the degree of dysarthria attributed in the perceptual analysis. 

 The worsened self-perception of speech in LOPD over time may be explained by a different natural course of LOPD, likely caused by the accelerated spread of neurodegenerative pathology in the elderly and the gradual decline of nigrostriatal dopaminergic neurons that naturally occurs during aging^1^. Furthermore, the increased severity of imprecise consonantal articulation and reduced voice quality in this group compared to EOPD, as reported by Ruzs, may be associated with more widespread neurodegeneration, which could also help explain our findings. Considering that LOPD presents with more expressed gait disturbances^31^, we may hypothesize that certain speech abnormalities will be detectable only in LOPD. In addition, several previous studies have reported a relationship between speech and gait disorders^32,33^. 

 A study carried out by Dias et al.^34^ identified that patients in moderate or advanced stages (average disease duration of ten years, average H&Y stage=3) had worse articulation—observed through perceptual-auditory and acoustic analysis—as the disease progressed, supporting the results of this study. All patients maintained speech intelligibility, but alterations in vowel articulation were detected by the vowel articulation index (VAI), indicating more significant articulatory impairment^34^. Our study found that patients in the LOPD group had longer syllabic duration, contributing to the worsening self-perception of speech. A slower articulation rate may lead to a perception of reduced speech fluency, increased effortfulness, and difficulty in maintaining natural prosody. This could influence how individuals with PD perceive their speech impairments. Rusz et al.^1^, when comparing EOPD and LOPD patients, identified that the latter group presented significantly shorter symptom duration, higher postural instability and gait difficulties (PIGD) score, a higher number of comorbidities, and overall higher severity of non-motor symptoms, including a higher prevalence of rapid eye movement sleep behavior disorder, lower Montreal Cognitive Assessement (MoCA) score, and higher Scales for Outcomes in Parkinson’s Disease - Autonomic Dysfunction (SCOPA-AUT) score. 

 We can hypothesize that the self-reported deterioration of speech in both groups with disease progression might result from a "calibration" deficit arising from a discrepancy between the sense of effort and the produced vocal intensity^35^. EOPD and LOPD patients seemed to be able to recognize and identify speech impairment associated with preserving central and peripheral sensorimotor feedback cycles^36^. Our study also observed this capability; however, our patients tended to decrease self-perception as the disease progressed. These patients tended to underestimate their speech difficulties over time, showing a reduced ability to recognize worsening speech features. Self-perception should not be considered in these patients, as it is not a reliable measure. Cognitive factors may have impacted the decline in self-perception, although they were not tested in our sample. We uncovered three phenotype-specific speech characteristics differing between EOPD and LOPD. Speech performance analysis based on respiration, phonation, articulation, prosody, and resonance was conducted to identify deviations in speech production, specifically evaluating articulatory precision and speech intelligibility. These parameters were assessed by speech therapists using auditory-perceptual rating that identify dysarthria subtypes and determine severity^2,28^. Despite similar perceptual dysarthria severity in both PD subgroups, EOPD showed weaker inspirations, while LOPD was characterized by decreased voice quality and imprecise consonant articulation. Also, this study highlighted three specific characteristics of hypokinetic dysarthria, including monopitch, monoloudness, and articulatory decay, that was consistently presented in both PD phenotypes and were unrelated to aging. 

 Patients with shorter disease duration and more severe dysarthria had higher self-perception of speech changes, suggesting that disease duration and overall dysarthria severity also impact self-perception. A slower articulation rate may lead to a perception of reduced speech fluency, increased effortfulness, and difficulty in maintaining natural prosody, which could influence how individuals with PD perceive their speech impairments. These communication deficits are a significant concern for individuals with PD as they impact their sense of acceptance, security and quality of life^21^, a finding that aligns with our study. 

 The fact that the brains of older patients with PD may have reduced compensatory mechanisms, leading to more pronounced impairment with faster disease progression affecting speech, supports our findings that showed worsened self-perception in LOPD patients with disease progression^1^. This study also emphasizes that the three specific characteristics of hypokinetic dysarthria, including monopitch, monoloudness, and imprecise articulation, were consistently presented in both phenotypes of Parkinson’s disease and were not related to aging. This allows us to hypothesize that speech changes in individuals with PD are more related to the time and progression of the disease than to age per sex. 

### Limitations and future perspectives

 The sample was not controlled for disease duration, so patients in the early-onset group had a longer disease duration than those in the late-onset group. This difference in disease duration could influence the outcomes and should be considered in future studies. Another limitation to be highlighted refers to the unequal distribution between the subgroups, especially with regard to the degree of dysarthria. For example, in the LOPD group, only three individuals presented severe dysarthria, which may limit generalization of the results to this specific profile. Future research should control for disease duration to better understand the differences between early- and late-onset PD. 

 In conclusion, the results between the clinical diagnosis of dysarthria and self-perception in early- and late-onset groups showed no significant differences, indicating that self-perception could be a useful tool for identifying cases, especially for mild dysarthria, for early intervention. However, in the group classified as severe dysarthria in LOPD individuals perceived themselves as less impaired. This discrepancy might be attributed to cognitive decline, which could be due to the disease, age, or both. 

## Data Availability

The datasets generated and/or analyzed during the current study are available from the corresponding author upon reasonable request.
